# Antiproliferative Activity on Human Colon Adenocarcinoma Cells and In Vitro Antioxidant Effect of Anthocyanin-Rich Extracts from Peels of Species of the *Myrtaceae* Family

**DOI:** 10.3390/molecules26030564

**Published:** 2021-01-22

**Authors:** Nayara Simas Frauches, Júlia Montenegro, Thuane Amaral, Joel Pimentel Abreu, Gabriela Laiber, Jorge Junior, Renata Borguini, Manuela Santiago, Sidney Pacheco, Vania Mayumi Nakajima, Ronoel Godoy, Anderson Junger Teodoro

**Affiliations:** 1 Laboratory of Functional Foods, Federal University of Rio de Janeiro State, Avenida Pasteur 296-Urca, 22290-240 Rio de Janeiro, RJ, Brazil; nanasimas@hotmail.com (N.S.F.); juliamontenegro95@gmail.com (J.M.); thuane.amarall@gmail.com (T.A.); pimenabreu@gmail.com (J.P.A.); gabrielalaiberpascoal@gmail.com (G.L.); 2 Federal University Fluminense, Mario Santos Braga Street, 30-Centro, 24020-140 Niteroí, RJ, Brazil; jorge.junior.pinho@gmail.com (J.J.); vania_nakajima@yahoo.com.br (V.M.N.); 3 Embrapa Food Technology, Avenida das Américas, 23020-470 Rio de Janeiro, RJ, Brazil; renata.borguini@embrapa.br (R.B.); manuela.santiago@embrapa.br (M.S.); sidney.pacheco@embrapa.br (S.P.); ronoel.godoy@gmail.com (R.G.)

**Keywords:** jabuticaba, malay-apple, jamun-berry, *Myrtaceae* fruits, colon cancer, bioactive compounds

## Abstract

There is a significant indication of the beneficial health effects of fruit rich diets. Fruits of native plant species have noticeably different phytochemicals and bioactive effects. The aim of this work was to characterize and compare the constituents of jabuticaba (*Myrciaria jaboticaba*, MJ), jamun-berry (*Syzygium cumini*, SC), and malay-apple (*Syzygium malaccense*, SM) extracts and their influence on antioxidant activity in vitro and antiproliferative effects on human colon adenocarcinoma cells. According to the results, dried peel powders (DP) have a high anthocyanin content, phenolic compounds, and antioxidant activity when compared to freeze dried extracts (FD). *M. jaboticaba* dried peel powder extract had a higher total anthocyanin and phenolic compounds content (802.90 ± 1.93 and 2152.92 ± 43.95 mg/100 g, respectively). A reduction in cell viability of HT-29 cells after treatment with *M. jaboticaba* extracts (DP-MJ and FD-MJ) was observed via MTT assay. Flow cytometry showed that the treatment with the anthocyanin-rich extracts from MJ, SC, and SM had an inhibitory impact on cell development due to G_2_/M arrest and caused a rise in apoptotic cells in relation to the control group. The findings of this study highlight the potential of peel powders from *Myrtaceae* fruits as an important source of natural antioxidants and a protective effect against colon adenocarcinoma.

## 1. Introduction

Fruits have different substances with antioxidant activity, such as polyphenols and carotenoids, which may reduce reactive oxygen species (ROS) levels in the human body, avoiding DNA damage and mutations [1,2]. The antioxidant capacity of a polyphenol can be credited to the reducing capacity of the aromatic hydroxyl (OH) group that causes a decrease in reactive free radicals [3].

Despite the expanding quantity of discoveries demonstrating that polyphenols exhibit antioxidant potential, the mechanisms by which they act goes beyond the modulation of oxidative stress [4]. A few reports reveal that polyphenol antioxidant activity is fully linked to the adjustment of mitochondrial function, expanding mitochondrial respiration, especially oxygen utilization driven ATP synthesis [5,6]. Polyphenols also have the capacity to increase the activity of phase II metabolizing enzymes acting in antioxidant responsive element pathways [7].

Among the polyphenols present in foods, anthocyanins may be highlighted, which are flavonoids responsible for the brilliant red to violet colors of fruits [8]. Although not considered a nutrient, anthocyanins have earned a lot of consideration due to their bioactive properties. [9].

In Brazil, we can find a variety of fruits rich in anthocyanins among the native and tropical species of *Myrtaceae* family as jabuticaba (*Myrciaria jaboticaba* (Vell) O. Berg), malay-apple (*Syzygium malaccense* (L.) Merr. and LM Perry), and jamun berry (*Syzygium cumini* (L.) Skeels). Their huge bioactive properties are most likely explained by the content of anthocyanins, mainly present in the peel. *M. jaboticaba* fruits are globular, with a whitish pulp and a peel that ranges from red to black, where their anthocyanins are concentrated. *S. malaccense* fruits are piriform and display a deep red color peel. *S. cumini* are small ovoid fruits that present purple peel and pulp color when ripe, indicating great anthocyanins content [10].

Colorectal cancer (CRC) is, globally, the third most frequent type of malignant tumor and the fourth most frequent mortality cause related to cancer [11,12]. Currently, more than 1 million new diagnoses of CRC are made annually [13]. CRC development is the result of the build-up mutations or epigenetic modifications that prompts the conversion of a standard colonic tissue layer into an adenocarcinoma. Several data in the literature point out the role of habitual diet as a significant cause in the development of CRC, and some dietary epidemiological researches have proposed that diets with lots of vegetables and fruits are rich in phytochemicals, which could be associated with a reduction in the chance of developing CRC [14,15].

Several models are used to study the association between diet and colon cancer. Critical alterations in the genetic expression of carrier proteins and metabolic enzymes in ordinary human enteral cells may influence the ability of the models to reproduce the permeability in vivo [16]. The HT-29 cell line was initially separated from a differentiated human adenocarcinoma and serves as a valuable in vitro framework for research on both expansion regulation and differentiation in colon cancer cells [17]. It is resistive to the actions of cytokines, TNF-α, and TNF-related apoptosis-promoting binders. In HT-29 cells, and similar enteral scrapings, comparable protein synthesis has been observed, some of which seem to be distinctive to the human colonic epithelium in vivo [18].

Few studies have associated fruit extracts of these Brazilian fruits rich in anthocyanins and with their role in the reduction of risk and in the treatment of chronic diseases, including cancer [8,19,20]. This protective role can be attributed to the biological effects of anthocyanins due to the antioxidant activity, antiproliferative, anti-mutagenic, and anti-carcinogenic functions. [21]. Anthocyanin-rich extract studies suggest they exert antiapoptosis and antiproliferative effects. Anthocyanins act as antiproliferative agents in vivo through upregulation of malignant cell apoptosis mechanisms [22]. Some studies have demonstrated downregulating pro-oncogenic signals and stimulating the expression of tumor suppressor genes, controlling proliferation and apoptosis pathways [23,24]. Thomasset et al. [25] showed tumor resection reduced the proliferation index in CRC patients through anthocyanins-rich extract (ARE) from bilberry treatment, explained by lower Ki-67 expression and an increased apoptotic index, observed by higher cleaved caspase-3 expression. Hence, the aim of this study was to identify and compare the extracts of three *Myrtaceae* Brazilian native fruits obtained by two different methods. These extracts were additionally investigated for their antiproliferative effect and apoptotic induction in HT-29 colonic cancer cells. The differential of the article was to address issues not yet discussed and identified the main phenolic compounds (anthocyanins) in three-selected species of Amazon and their interaction between the antioxidant proprieties and antiproliferative activity in colon cancer.

## 2. Results and Discussion

### 2.1. Color Analysis

Cartesian polar coordinates describe the CIELAB color space. The L* hub spreads from the top to the base, and the utmost value is characterized by 100 (denotative of color), whereas the lowest is 0, which represents no light. The coordinate a* shows a range from green to red shading (green is represented from −80 to zero, red is indicated from zero to +100), whilst the b* coordinate illustrates the discrimination of blue and yellow depth (blue is indicated from −100 to 0, yellow is characterized from 0 to +70) [26,27].

It was observed that the extracts of *M. jaboticaba* (MJ) presented a lower value of L* in comparison to *S. cumini* (SC) and *S. malaccense* (SM) (Table 1). Lower values of L* in jabutibaca extracts were desirable since they indicate the effectiveness of the anthocyanin extraction. Regarding the parameter a*, it was observed that *S. malaccense* extracts (Table 1) presented with higher values referring to the red coordinate compared to other fruits [28]. *S. malaccense* peel showed an intense coloration, ranging from pink, alizarin, and deep red shades, according to the maturation stage or crop state [29].

*S. cumini* samples presented with lower values for the coordinate b* that assigns yellow coloration. This result can be justified since the peels of *S. cumini* present a characteristic dark coloration that has a greater amount of dark pigments coming from anthocyanins, which could have contributed to the reduction of this parameter [30,31].

### 2.2. Phenolic Compound Content

**Figure 1:**
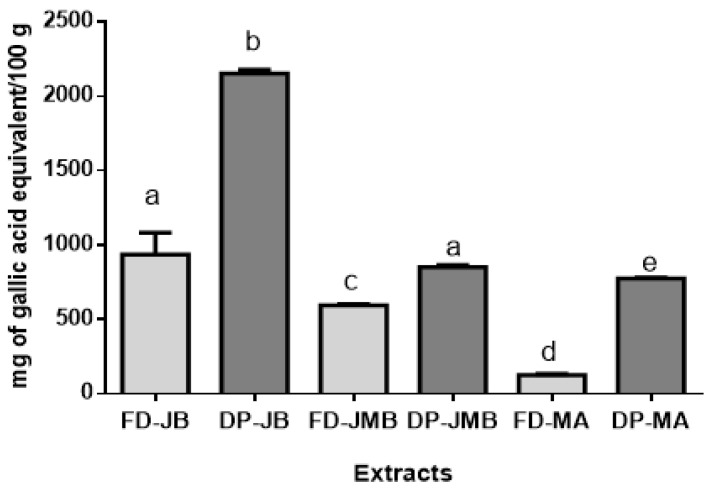
Total phenolic compounds of freeze-dried (FD) and dried peel powders (DP) extracts of *M. jaboticaba* (FD- MJ and DP-MJ), *S. cumini* (FD-SC and DP-SC), and *S. malaccense* (FD-SM and DP-SM). Results are expressed as mean ± standard deviation; means with different letters in the same line are significantly different (*p* < 0.05, Tukey’s test).

The Folin–Ciocalteu method is related to the reducing capacity of phenolic compounds, and the results obtained can be observed in Figure 1. Phenolic compound analysis revealed that higher values were related to *M. jaboticaba* (Figure 1). The mean value presented in the FD-MJ sample was 1190 ± 9.48 mg EAG/100 g, followed by the DP- MJ sample with 2149.58 ± 6.89 mg EAG/100 g. FD-MJ extract showed a lower content of total phenolic compounds in relation to the DP-MJ.

Although most phenolic compounds have polar characteristics and are therefore compatible with the aqueous extractor, the lowest concentration found in the freeze-dried aqueous extract can probably be due to the fact that anthocyanins are usually bonded to insoluble compounds, such as fibers. This would reduce availability and extraction efficiency and resulted in a lower concentration of these compounds in the final product when compared to the dried powder, which is the peel directly dried and have all the phytochemicals present in the fruit’s peel [32,33].

Leite-Legatti et al. [8] found mean quantities for total phenolic compounds of 556.3 mg EAG/100 g in *M. jaboticaba′s* peel. Reynertson et al. [34], after analysis of *M. jaboticaba* extract, observed a mean content of 31.6 mg EAG/100 g. Previously, it has been shown that a high content of phenolic compounds of fruits can effectively act to reduce the risk of cancer development [35,36]. In this context, the consumption of individual sorts of phenolic compounds is likely beneficial for human health [37]. These fruits have important phenolic compound content, and therefore, are probably beneficial to health.

### 2.3. Anthocyanin Quantification

Table 2 displays the data acquired in high-performance liquid chromatography (HPLC) analyses of anthocyanin-rich extracts. Only the major anthocyanins (cianidin-3,5-*O*-diglucoside, cianidin-3-*O*-glucoside, delfinidin-3-*O*-diglucoside, delfinidin-3,5-*O*-glucoside, petunidin-3,5-*O*-diglucoside, and malvidin-3,5-*O*-diglucoside) were quantified in this analysis. *M. jaboticaba* (Figure S1), *S. cumini* (Figure S2), and *S. malaccense* (Figure S3) peels are sources of anthocyanins, since fruits considered to be a source are those that present more than 2 mg/g of anthocyanins [38,39].

In freeze-dried samples, *S. cumini* had higher total anthocyanin content (231.03 ± 0.32 mg/100 g) due to a greater variety of anthocyanins in its composition. *S. cumini* also displayed purple pigments in the pulp, not only in the peel, as in the other two species. This may indicate that its anthocyanins are more accessible because it may not be entirely bonded to fibers from the peel and would be extracted more easily [33].

Regarding the samples of dried peel powder, it was verified that *M. jaboticaba* presented the highest total anthocyanin content (802.90 ± 1.93 mg/100 g), and it was identified and quantified delphinidin-3-*O*-glucoside and cyanidin-3-*O*-glucoside. These findings support the data present in the literature [31,40,41].

There are few studies in the literature that exhibit the anthocyanins profile of *M. jaboticaba*, *S. malaccense*, and *S. cumini* by HPLC with mass spectroscopy (MS). It has been revealed the mean value of cyanidin 3-glucoside in the freeze-dried fruit for *M. jaboticaba* peel was 2.78 mg/g, 6.33 mg/g for *S. cumini*, and trace (below 0.01 mg/g) for *S. malaccense* [33]. The average content of monomeric anthocyanins was described as 12.90 mg/g in the fresh pulp of the *S. malaccense* [29]. However, to the best of our knowledge, a characterization of anthocyanins profile of these fruits’ peels has not yet been made.

### 2.4. Antioxidant Activity

The antioxidant capacity analysis was performed by different methods (2,2-diphenyl-1-picrylhydrazyl (DPPH), Ferric Reducing Ability (FRAP), Trolox Equivalent Antioxidant Capacity (TEAC), and Oxygen Radical Absorbance Capacity (ORAC)) for a better understanding of the data (Table 3). Because of the various sorts of free radicals and their diverse means of action in living organisms, there is hardly a simple and universal assay that can measure exactly and quantitatively the antioxidant capacity. Hence, a single analysis would be insufficient to evaluate antioxidant activity [42,43,44].

Table 3 shows that higher values were obtained by the DP-MJ samples, followed by DP-SC and FD-SC. These data are in agreement with the previous results of total phenolic compounds. It was expected that samples with higher concentrations of phenolic compounds would present higher antioxidant activity.

Higher antioxidant values were obtained using the TEAC method, followed by ORAC, FRAP, and DPPH assays, respectively. The method for measuring phenolic compound is based on reducing capacity, and so is FRAP assay; thus, these methods are more related. DPPH, TEAC, and ORAC are based on free radical scavenging capacity but differ in compatibility with different types of compounds.

Also, Table 3 reveals that the dried peel powder samples presented with higher values of antioxidant activity compared to freeze-dried. In this study, the reduction of antioxidant activity was directly associated with phenolic compounds and anthocyanins, indicating that these substances are possibly responsible for the antioxidant potential.

Based in these findings, *Myrtaceae* fruit peels may show potential antioxidant capacity and may be considered a valuable source of natural antioxidants, preventing the harmful effect of free radicals. Free radicals and reactive oxygen species (ROS) have been considered to contribute to the progress of aging and illness by causing oxidative stress [45,46]. Aimed at improving health, the consumption of these natural sources of substances can be vitally important to the human body. Phenolic compounds and anthocyanins contribute to the maintenance of the pro and antioxidant balance of biological systems [20].

### 2.5. Cell Assays Results

#### 2.5.1. Effect of *M. jaboticaba* (FD-MJ and DP-MJ), *S. cumini* (FD-SC and DP-SC), and *S. malaccense* (FD-SM and DP-SM) Extracts on Cell Viability

**Figure 2:**
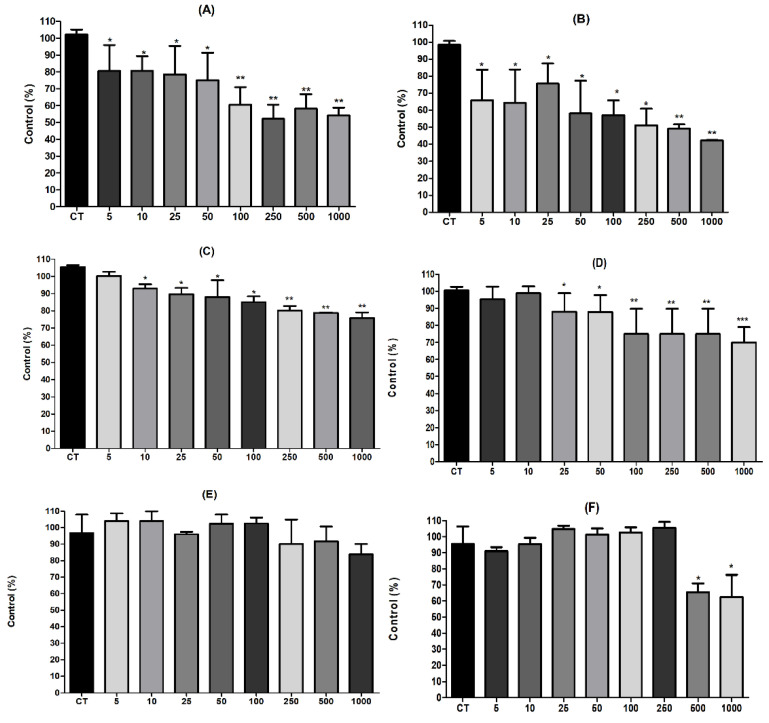
Effect of freeze-dried (FD) and dried peel powders (DP) extracts of *M. jaboticaba* FD-MJ (**A**) and DP-MJ (**B**), *S. cumini* FD-SC (**C**) and DP-SC (**D**) and *S. malaccense* FD-SM (**E**) and DP-SM (**F**) on viability HT-29 cells after 24 h of treatment (5–1000µg/mL). The results were compared by one-way ANOVA with the post-test of Tukey’s test (* *p* < 0.05; ** *p* < 0.01; *** *p* < 0.001).

The anthocyanins rich extracts decreased the number of viable HT-29 cells within 24 h (Figure 2). The *M. jaboticaba* samples (FD-MJ and DP-MJ) caused the highest decrease in viability compared to control (45.86% and 57.77%) at the concentration of 1000 μg/mL, while cells exposed to jamum berry samples (FD-SC and DP-SC) had a reduction in cell viability reduction of 24.17% and 30%, at a concentration of 1000 μg/mL. *S. malaccense* extract (FD-SM) caused a lower reduction in cell viability (16.08%) and reduced viability by 38% (1000 μg/mL). These results are in agreement with the results found in total phenolic compound and antioxidant activity analyses, indicating that these activities are correlated.

As shown in Figure 2, a slight decrease was observed in lower concentrations of the extracts rich in anthocyanins, evidencing a small non-significant alteration in the viable cell growth profile. Anthocyanins have been demonstrated to have some biofunctional activities such as chemoprevention and apoptosis induction. Besides that, the anti-cancer deeds of anthocyanins have been described in vitro and in vivo, concerning the prevention of oncogenesis and tumor infiltration [47,48,49]. Furthermore, *M. jaboticaba* extracts have conferred an IC_50_ value for VERO non-tumoral cell lineage above than that determined for the most susceptible cells, which may indicate low toxicity to regular cells [8].

Few studies report the influence of *Myrtaceae* fruits on the antiproliferative effects on cancer cells. It has been reported that several flavonoid compounds from *M. cauliflora* presented antiproliferative activities against HT-29 (IC_50_ = 65 μM) and HCT116 (IC_50_ = 30 μM) colon cell lines [45]. Also, there are some findings concerning *S. cumini* extracts that reported the inhibition on the colon cancer line (HCT116). The extract of *S. cumini* showed a reduction of approximately 50% in relation to the viable cells in both concentrations (30.0 μg/mL and 40.0 μg/mL) [50]. Furthermore, Rabeta et al. [51] evaluated the action of jambo methanolic extracts and found a significant antiproliferative effect with 79% viability cells in MCF-7 breast cancer (IC_50_ = 632.3 μg/mL).

Our work provides several important data on the antiproliferative action of fruit peel extracts in colon cancer. Although our model was performed with only the HT-29 cell line, these cells have some peculiar characteristics important in the treatment of cancer. HT-29 in its differentiated phenotype resembles small intestine enterocytes regarding its morphology, the existence of hydrolases related to the brush border, and the differentiation process time course. Also, the quantity of villin presented in differentiated HT-29 cells is near to the value determined for typical just disposed colonocytes.

Additionally, the HT-29 cell line is acquiring specific importance in research focused on nutrients assimilation and bioavailability due to the capacity to present characteristics of developed enteric cells [52,53].

#### 2.5.2. Effect of *M. jaboticaba* (DP-MJ), *S. cumini* (DP-SC), and *S. malaccense* (DP-SM) Extracts on the Cell Cycle

**Figure 3:**
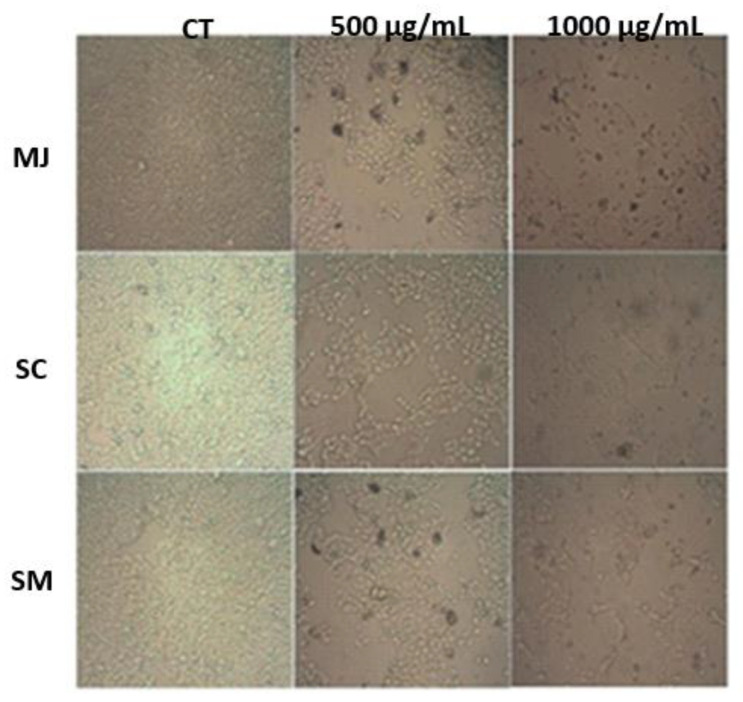
Photomicrographs from phase-contrast microscopy of HT-29 cells treated with dried peel powders (500 and 100 µg/mL) extracts of *M. jaboticaba* (MJ), *S. cumini* (SC), and *S. malaccense* (SM). Phase-contrast images were taken from random fields (80 µm).

The regulation of the cell cycle is an essential matter in cancer treatment [54]. Based on phenolic compounds, antioxidant activity, and cell viability assays, the dried peel powder samples were chosen to be tested on the HT-29 cell cycle. In this scenario, the dried powder of *M. jaboticaba*, *S. cumini,* and *S. malaccense* demonstrated a capacity to restrain the proliferation of HT-29 cells (Figure 3).

To scan the inhibition of cell growth, mediated by DP-MJ, DP-SC, and DP-SM, the cell cycle was examined by flow cytometry. The percentage of cells in each phase of the cell cycle are shown in Table 4.

After 24 h treatment with DP-MJ, we found an influence on HT-29 cells, with a significant decrease of G_2_/M phase, that reached 32% at a 1000 μg/mL concentration, which can be explained by the agglomeration of cells on G_0_/G_1_. Similarly, the DP-SC and DP-SM extract promoted an arrest in G_0_/G_1_ stage, followed by a reduction in G_2_/M stage population of cells. In addition, all fruit extracts (DP-MJ, DP-SC, and DP-SM) prompted a collection of cells at the S stage.

DP-MJ, DP-SC, and DP-SM extracts contain cyanidin-3-*O*-glucoside, delphinidin-3-*O*-glucoside, and petunidin-3,5-*O*-diglucoside. It was reported that a breast cancer cell line (Hs578T) was susceptible to cyanidin 3-glucoside, as well as the therapy using this anthocyanin caused a substantial repressive impact on cell development by means of G_2_/M suppression through induced caspase-3 activation, chromatin condensation, and cell death [55].

Also, pomegranate extract, which contains six anthocyanins (pelargonidin-3-*O*-glucoside, cyanidin-3-*O*-glucoside, delphinidin-3-*O*-glucoside, pelargonidin-3,5-*O*-diglucoside, cyanidin-3,5-*O*-diglucoside, and delphinidin-3,5-*O*-diglucoside), was capable of limiting prostate cancer cells (LAPC4) development, through CKI-cyclin-CDK network adjustment, with up-regulation of p21 and p27 throughout restraining to the G1-stage, irrespective of p53 [56].

Another study reported that anthocyanins rich extracts from blueberry, grape, and aronia were efficient to considerably repress the development of HT-29 cells, with a small impact on normal colonocytes development (NCM460) [57,58]. This investigation demonstrated that anthocyanins-rich extract (50 µg/mL) restrained development and cell cycle process in a double blockage at G_1_/G_0_ and G_2_/M stages on colon adenocarcinoma cells mainly due to up-regulation of p21WAF and p27 kip1, and down-regulation of cyclin A and cyclin B1 genes [57].

In this way, our results show that anthocyanins rich extracts of DP-MJ, DP-SC, and DP-SM were able to modify cell cycle, showing that the extracts studied may be promising anti-carcinogenic or chemoprotective agents for additional examination.

#### 2.5.3. Apoptosis

The effects of DP-MJ, DP-SC, and DP-SM extracts in HT-29 cells were examined for apoptotic death. The proportion of viable, non-apoptotic, early apoptotic, and late apoptotic cells in relation to 500 and 1000 µg/mL treatment are shown in Table 5.

**Figure 4:**
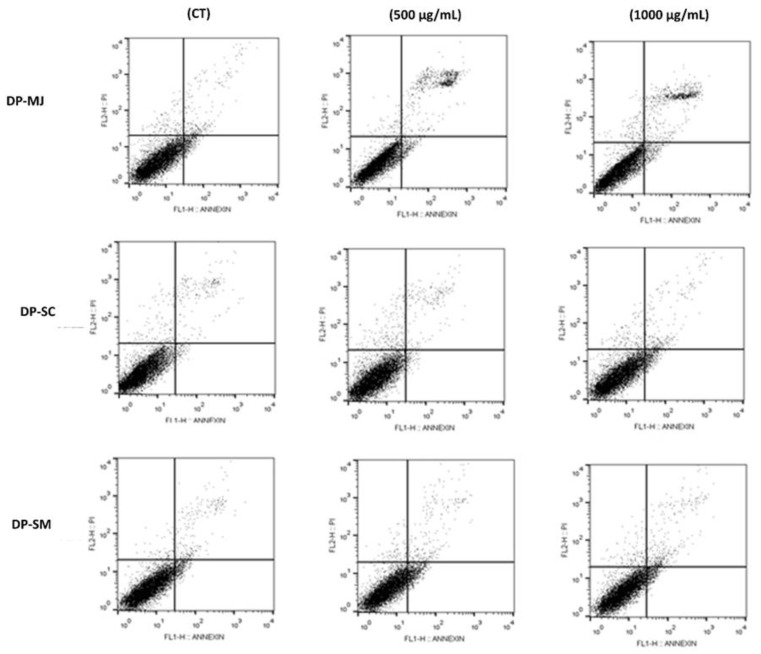
Effect of dried peel powders (DP) extracts of *M. jaboticaba* (MJ), *S. cumini* (SC), and *S. malaccense* (SM) on the process of programmed death in HT-29 cells after treatment for 24 h. Flow cytometry analysis according to the exposure time and extracts concentration (500 and 1000 µg/mL).

Figure 4 shows the impact of DP-MJ, DP-SC, and DP-SM extracts on the rate of apoptosis. Following 24 h, cells treated with DP-MJ (500 and 1000 µg/mL) resulted in a 2.74- and 2.29-fold increase in the level of both early and late apoptotic cells, respectively, compared with untreated cells (control). This effect was followed by a decline in the number of HT-29 viable and non-apoptotic cells. In addition, the high concentrations of DP-MJ (500 and 1000 µg/mL) showed little effect on the percentage of non-apoptotic cell death, possibly indicating low toxicity in the extracts. No significant variation in apoptosis induction between DP-SC and DP-SM and controls was noticed (Figure 4 and Table 5).

Expanded resistance to apoptosis is characteristic of different types of cancers. The functional suppression of anti-apoptotic elements could offer a reasonable basis for the improvement of new treatment methodologies. Thus, flaws in apoptosis regulation are viewed as a serious reason behind tumors’ treatment resistance because several bioactive compounds operate in inducing apoptosis.

Wang et al. [59] analyzed the impact of the aqueous extract from different parts of *M. jaboticaba* on an oral cancer cell line (HSC-3). The aqueous extract of the seed at the concentration of 50 μg/mL increased the apoptosis rate, presenting the value of 57.1% in relation to untreated cells. In another study, anthocyanin-rich extracts of grape and strawberry and their produced metabolites, like hydroxyphenylacetic acid, demonstrated apoptotic action in HT-29 cell line after 8 h of treatment with annexin V, and proposes their potential collaboration as protectors against cancer [60]. Blueberry dried extracts with a high number of anthocyanins were reported to provoke apoptosis in HT-29 and Caco-2 cells and caused a two to seven-fold rise in DNA fragmentation [61].

The apoptosis process is characterized by a scheduled sequence of events that leads to the elimination of cells without causing damages to the adjacent tissues. This process, which causes distinct changes in cell morphology, is responsible for keeping cells healthy by eliminating excess abnormal cells [62]. One of the common features of cancer is the avoidance of cell death and portrays a significant source of resistance to typical therapeutic techniques. Thus, the capacity of a compound to restrain the expansion of cancer cells is highly desirable [63,64]. However, the apoptotic action of the studied fruits has not been widely recorded in the literature.

## 3. Materials and Methods

### 3.1. Chemicals

Acetonitrile, formic acid 98%, methanol, and ethanol were obtained from Tedia (Fairfield, OH, USA). The ultrapure water was produced using Milli-Q Gradient 10A System (Merck Millipore Corporation, Burlington, MA, USA). The delphinidin-3,5-diglucoside chloride standard was bought from ChromaDex TM (Los Angeles, CA, USA). Cyanidin 3-glucoside was purchased from Indofine Chemical Co. (Somerville, NJ, USA). DPPH (2,2-diphenyl-1-picrylhydrazyl), ABTS (2,2-azinobis(3-ethylbenzo-thiazoline-6-sulfonic acid)) and Trolox (6-Hidroxi-2,5,7,8-tetrametilchroman-2-carboxylic acid) were acquired from Sigma-Aldrich (St Louis, MO, USA). AAPH (2,2′-azobis ((2-methylpropionamidine) dihydrochloride); Fluorescein; Dulbecco’s cell culture medium and bovine serum albumin were purchased from Sigma. Serum fetal bovine and MTT (3-(4,5-dimethylthiazol-2-yl)-2,5-diphenyltetrazolium bromide) were acquired from Laborclin (Campinas, Brazil). All the chemicals had an analytical and HPLC grade.

### 3.2. Anthocyanin-Rich extracts

#### 3.2.1. Samples

*S. malaccense* and *S. cumini* samples were collected in the Guaratiba district (Rio de Janeiro, Brazil), and the *M. jaboticaba* samples were obtained directly from the producer on a farm located in the region of Joaquim Egídio (Campinas, São Paulo, Brazil). 

#### 3.2.2. Dried Peel Powder and Freeze-Dried Extracts

For the preparation of dried powder of *M. jaboticaba* (DP-MJ), *S. cumini* (DP-SC), and *S. malaccense* (DP-SM), the fruits were cleaned, and the peel was manually isolated from the pulp. On the same day, the peel was subjected to a drying procedure. The drying method was handled on a convective layer dryer created by Embrapa Food Technology. The peels were set in place on boards in individual layers and exposed to drying out at 60 °C and an air speed of 1 m/s for 20 h. The dehydrated yield was triturated utilizing a blender and stocked in aluminum and polyethylene packs at room temperature until analysis [31]. For the preparation of freeze-dried, the samples (250 g/L) were subjected to an aqueous extraction procedure and lyophilized for 24 h. The freeze-dried samples were stored in the falcon tube and were frozen at −18 °C until analysis.

### 3.3. Color Analysis

The colorimetric test was performed in triplicate using a colorimeter (Color Quest XE), and the CIELAB scale with a 0.375 mm gap width, and D65/10 lighting. The observation angle was 10 mm quartz cuvette. From the values of the parameters L* (lightness), a* and b* color coordinates, the colors were determined [26].

### 3.4. Total Phenolics Content

Total phenolic compounds assay was carried out using the Folin–Ciocalteu method [65]. The Folin–Ciocalteu reagent (10%) was added to aliquots of each sample, completely diluted in distilled water (250, 500, and 1000 μL), and after 5 min sodium carbonate 4% was added. A gallic acid standard curve was made, and after 1 h in the dark, they were measured in 760 nm. The results are presented in mg of gallic acid equivalent (GAE)/100 g of the sample.

### 3.5. Anthocyanins Analysis

#### 3.5.1. Sample Extraction

From 1 g of each anthocyanin-rich extracts, 10 mL of methanol/formic acid solution (90:10 *v*/*v*) was used for the extraction process. The samples were vortex mixed for 1 min and sonicated for 10 min at 20 °C and were centrifugate for 10 min. The supernatant was decanted to a collection vial, and the samples were extracted three more times with 2 mL of methanol/formic acid solution. The combined extract was diluted to 10 mL with methanol/formic acid solution (90:10 *v*/*v*) prior to chromatographic analysis [66].

#### 3.5.2. HPLC with MS

The tests were performed in triplicate on a WatersTM Alliance 2695 system, WatersTM 2996 photodiode array (at 520 nm), and a Rheodyne^®^ six-channel selection valve. The column used was a ThermoTM Scientific C18 BDS (100 mm × 4.6 mm; 2.4 µm). The mobile phase consisted of 10% aqueous formic acid (solvent A) and methanol (solvent B). A gradient elution method with acetonitrile and formic acid was used. An external standard curve of cyanidin 3-glucoside was used, based on calibration curves prepared with HPLC analytical standards produced in Embrapa Food Technology, with purity higher than 99% and confirmed with mass spectrometry of high resolution. The selecting valve was programmed to switch to channel one at the beginning of the cyanidin-3-*O*-glucoside elution (at 16.2 min) and switch back to discharge position after its partial elution (at 18.4 min) column, a flow of 1.0 mL/min, column temperature of 40 °C, injection volume of 20 µL. The results show the total anthocyanins content, and the concentrations have been expressed as cyanidin 3-glucoside equivalents. The quantification was performed using the Agilent Chemstation software (Agilient Technologies, Santa Clara, CA, USA) [66,67,68].

### 3.6. Antioxidant Activity

#### 3.6.1. DPPH Assay

Aliquots (300 μL) of the extracts diluted in distilled water were blended with 2.2 mL DPPH methanolic solution (0.06 mM) and kept in the dark for 1 h. Readings were determinate at 515 nm using a Turner 340 spectrophotometer. The assay was performed in triplicate. The antioxidant activity was calculated from the equation obtained by the linear regression after plotting known concentration solutions of Trolox. The results were presented as μmol Trolox equivalents/g dry basis [69].

#### 3.6.2. Trolox Equivalent Antioxidant Capacity (TEAC)

The antioxidant activity analysis was performed by the adapted TEAC method [69]. The TEAC^+^ ion was set up by blending a TEAC stock solution to a 2.45 mM K_2_S_2_O_8_. This blend reacted for 16 h at room temperature. Then it was diluted in ethanol until it reached an absorbance of 0.700 ± 0.010 at 734 nm. Aliquots of 5, 10, and 20 µL of the extracts were tested, and a standard curve (Trolox) was made. Measurements were annotated using a spectrophotometer after 6 min of reaction. Antioxidant capacity was shown in µmol Trolox/g dry basis.

#### 3.6.3. Ferric Reducing Ability (FRAP)

A FRAP assay was performed in agreement with Thaiponga et al. [70]. Aliquots of 2.7 mL of FRAP solution (FeCl_3_, TPTZ, and acetate buffer) and 0.5 mL of samples were mixed. The absorbance was measured at 595 nm, after 30 min at 37 °C temperature. A standard curve was made using ferrous sulfate, and antioxidant activity was declared as µmol of Fe^2+^ equivalents/g dry basis.

#### 3.6.4. ORAC Assay

The Oxygen Radical Absorbance Capacity (ORAC) method was performed according to Prior et al. [71]. Shortly, samples were diluted in potassium phosphate buffer (pH 7.4) and plated in triplicate in a black 96-well microplate. Fluorescein (120 μL) was added to each well and incubated at 37 °C for 20 min, with discontinuous agitation, prior to the inclusion of 10 μL of recently prepared AAPH. The microplate was promptly placed into the fluorimeter (Thermo Labsystems). The decline of fluorescence was estimated at 538 nm emission with 485 nm excitation, every 30 s for 3 h. The samples’ area under the curve less the blanks were contrasted with a Trolox standard curve. Results were presented as µmol Trolox equivalents/100 g of fruit.

### 3.7. Cell Culture and Treatment Protocol

Certified human colon adenocarcinoma cell line (HT-29) was acquired from the Rio de Janeiro Cell Bank (Inmetro, Rio de Janeiro, Brazil). HT-29 cells were grown in cell culture bottles and cultivated regularly under 5% CO_2_ atmosphere, in DMEM, with 10% fetal bovine serum (FBS), 1% Penicillin (PS), and 0.2% HEPES buffer (pH 7.4). Stock cultures in flasks were grown to 80% confluence and routinely subcultured. Cell morphology was observed using a Zeiss Observer Z1 microscope, and all images were captured using Axio-Vision Rel. 4.8 software (Carl Zeiss, Jena, Germany).

### 3.8. Cell Viability

Cell viability was evaluated using MTT assay, as formerly reported [72]. Shortly, cells were plated in 96-well (5 × 10^3^ cells/well) and incubated for 24 h with or without 5–1000 µg/mL solution of fruit extract. Then, 10 μL of MTT (3-(4,5-dimethylthiazol-2-yl)-2,5 diphenyl tetrazolium bromide) was added to the culture plates, and the blue color of formazan produced by mitochondrial succinate dehydrogenase was analyzed in an enzyme-linked immunosorbent micro-plate reader (POLARIS-CELER^®^, Celer Biotecnologia, Minas Gerais, BH, Brazil) at 570 nm. Cell survival percentage was calculated using the mean of triplicate experiments compared to the mean control value.

### 3.9. Cell Cycle Analysis

Cells were gathered in logarithmic growth phase at a concentration of 1 × 10^6^ cells/mL. Different concentrations of fruit extract that have shown a reduction in cell viability (500–1000 μg/mL) were added to the cells. After 24 h of treatment, the floating and attached cells were caught, centrifuged, and rinsed with cold PBS. A fluorochrome solution including 50 μg/mL propidium iodide (PI), 3.4 mmol/L sodium citrate, 20 μg/mL RNase A, and 1% Triton X 100 were combined, and the blend was incubated for 30 min, in the dark at room temperature. The partition of the cell cycle was defined in a flow cytometer (FACSCalibur flow cytometer, Becton Dickinson, Mountain View, CA, USA). The test was operated using Cell Quest software (Beckton Dickinson and Company, Franklin Lakes, NJ, USA), and the percentage of cell community at a specific phase was calculated using the FlowJo software following the acquisition of 30,000 events.

### 3.10. Apoptosis Assay

Cells were plated in 6-multiwell plates (1.0 × 10^4^ cells/cm^2^) with culture medium, and after 24 h were incubated with (500 and 1000 μg/mL) DP-MJ, DP-JMA, and DP-MA for 24 h. To evaluate the proportion of apoptosis, the cells were subjected to coloring with Annexin V conjugated to FITC (BD Pharmingen, San Diego, CA, USA). The nonadherent cells were picked, and adherent cells were quickly rinsed with buffered saline solution (PBS) calcium/magnesium-free and were detached with trypsin/EDTA 0.125% (Sigma chemical Co., St. Louis, MO, USA) at room temperature. Afterward, apoptotic and necrotic cells were colored with Annexin V FITC/propidium iodide (PI) (BD Pharmingen, San Diego, CA, USA) in accordance with the producer’s instructions, quantified by flow cytometer (FACSCalibur, BD Bioscience, San Jose, CA, USA), and analyzed with FlowJo software.

### 3.11. Statistical Analysis

The outcomes are expressed as means with the analogous standard deviation of 2 independent experiments done in triplicates (*n* = 6). Data were evaluated with the statistical software GraphPad Prism (version 5.04, GraphPad Software, San Diego, CA, USA) and Statistica (version 7.0, StatSoft Inc., Tulsa, OK, USA). One-way analysis of variance (ANOVA) and Tukey’s test at a confidence level of 95% were applied.

## 4. Conclusions

The results obtained indicate that the important bioactive potential of *M. jaboticaba*, *S. cumini*, and *S. malaccense* is related to their anthocyanins and phenolic content. These species showed, in vitro, high antioxidant and total phenolic content, especially the dried powder peel ones.

The data from this work revealed that the dried peel powders of *M. jaboticaba*, *S. cumini*, and *S. malaccense* inhibited cell proliferation, arrest cell cycle, and raised apoptosis induction in human adenocarcinoma cells (HT-29) in a dose-dependent manner. Therefore, further studies are needed to clarify the putative therapeutic potential of these fruit extracts in colon cancer cells.

To date, studies reported in the literature about *M. jaboticaba, S. cumini*, and *S. malaccense* extracts are still scarce, especially about their influence on colon cancer cell lines. Our study is one of the first to provide experimental evidence that *M. jaboticaba*, *S. cumini*, and *S. malaccense* fruit peel extracts can inhibit the cell viability of HT-29 colon cancer cells. Studies with these fruit extracts reported on other cell lines have shown that they are potent antioxidants and provide early evidence that can be used to develop new chemotherapy strategies aimed at preventing the development of many diseases, including cancer.

## Figures and Tables

**Table 1:** Color analysis of freeze-dried (FD) and dried peel powders (DP) extracts of *Myrciaria* *jaboticaba* (DP-MJ and FD-MJ), *Syzygium* *cumini* (DP-SC and FD-SC), and *S. malaccense* (DP-SM and FD-SM).

Parameters	Freeze-Dried Extracts			Dried Peel Powder Extracts		
	FD-MJ	FD-SC	FD-SM	DP-MJ	DP-SC	DP-SM
L*	32.46 ± 0.01 ^a^	43.91 ± 0.04 ^b^	53.21 ± 0.01 ^c^	19.12 ± 0.02 ^d^	21.93 ± 0.03 ^e^	34.21 ± 0.03 ^f^
a*	14.67 ± 0.02 ^a^	18.32 ± 0.01 ^b^	20.84 ± 0.01 ^c^	8.15 ± 0.01 ^d^	11.19 ± 0.02 ^e^	15.91 ± 0.01 ^f^
b*	4.98 ± 0.02 ^a^	2.56 ± 0.03 ^b^	9.65 ± 0.02 ^c^	3.03 ± 0.01 ^d^	1.59 ± 0.01 ^e^	7.59 ± 0.01 ^f^

Results are expressed as mean ± standard deviation; ^a,b,c,d,e,f^: means with different letters in the same line are significantly different (*p* < 0.05, Tukey’s test).

**Table 2:** The anthocyanins concentration on freeze-dried (FD) and dried peel powders (DP) extracts of *M. jaboticaba* (FD- MJ and DP-MJ), *S. cumini* (FD-SC and DP-SC), and *S. malaccense* (FD-SM and DP-SM).

Anthocyanins (mg/100 g)	Freeze-Dried Extracts			Dried Peel Powder Extracts		
	FD-MJ	FD-SC	FD-SM	DP-MJ	DP-SC	DP-SM
Cyanidin-3,5-*O*-diglucoside	ND	9.75 ± 2.37 ^a^	8.46 ± 2.06 ^b^	ND	11.18 ± 2.10 ^a^	11.37 ± 2.12 ^a^
Cyanidin-3-*O*-glucoside	171.39 ± 4.22 ^a^	ND	61.87 ± 5.78 ^b^	789.48 ± 3.98 ^c^	ND	144.68 ± 5.52 ^d^
Delphinidin-3-*O*-glucoside	9.30 ± 1.26 ^a^	ND	ND	13.42 ± 1.93 ^b^	206.26 ± 1.83 ^c^	ND
Delphinidin-3,5-*O*-diglucoside	ND	51.37 ± 6.35	ND	ND	ND	ND
Petunidin-3,5-*O*-diglucoside	ND	86.90 ± 9.24 ^a^	ND	ND	208.26 ± 2.70 ^b^	ND
Malvidin-3,5-*O*-diglucoside	ND	83.01 ± 8.17 ^a^	ND	ND	149.50 ± 6.44 ^b^	ND
Total	180.69 ± 0.78 ^a^	231.03 ± 0.32 ^b^	70.33 ± 0.72^c^	802.90 ± 1.93 ^d^	575.20 ± 0.98 ^e^	156.05 ± 2.35 ^f^

Results are expressed as mean ± standard deviation; ^a,b,c,d,e,f^: means with different letters in the same line are significantly different (*p* < 0.05, Tukey’s test). ND—not detectable.

**Table 3:** Antioxidant activity of freeze-dried (FD) and dried peel powders (DP) extracts of *M. jaboticaba* (FD-MJ and DP-MJ), *S. cumini* (FD-SC and DP-SC), and *S. malaccense* (FD-SM and DP-SM).

Assays	Freeze-Dried Extracts			Dried Peel Powder Extracts		
	FD-MJ	FD-SC	FD-MA	DP-MJ	DP-SC	DP-MA
DPPH (µmol trolox/g)	554.44 ± 2.68 ^a^	324.93 ± 1.22 ^b^	61.24 ± 0.27 ^c^	576.02 ± 3.66 ^d^	378.46 ± 1.07 ^e^	100.27 ± 1.22 ^f^
FRAP (µmol Fe^3+^/g)	338.14 ± 3.15 ^a^	154.74 ± 3.51 ^b^	119.03 ± 2.29 ^c^	708.48 ± 3.40 ^d^	702.52 ± 2.79 ^d^	609.23 ± 3.51 ^e^
TEAC (µmol trolox/g)	987.15 ± 0.70 ^a^	1095.63 ± 3.76 ^b^	357.85 ± 1.00 ^c^	1271.91 ± 5.02 ^d^	1038.50 ± 2.98 ^e^	492.03 ± 3.99 ^f^
ORAC (µmol trolox/g)	508.81 ± 2.73 ^a^	241.92 ± 4.51 ^b^	21.23 ± 3.09 ^c^	883.94 ± 5.03 ^d^	610.23 ± 1.29 ^e^	570.18 ± 3.99 ^f^

Results are expressed as mean ± standard deviation; ^a,b,c,d,e,f^: means with different letters in the same line are significantly different (*p* < 0.05, Tukey’s test). TEAC—Trolox equivalent antioxidant capacity; ORAC—oxygen radical absorbance capacity. DPPH—2,2-diphenyl-1-picrylhydrazyl, FRAP—Ferric Reducing Ability.

**Table 4:** Effect of dried peel powders (DP) extracts of *M. jaboticaba* (DP-MJ), *S. cumini* (DP-SC), and *S. malaccense* (DP-SM) (500–1000 µg/mL) on cell cycle progression in HT-29 cells after 24 h.

	Cell Cycle Phase	CT	500 µg/mL	1000 µg/mL
	G_0_/G_1_	51.32 ± 0.03	51.56 ± 2.82	56.63 ± 2.31 *
DP-MJ	S	7.70 ±0.46	11.97 ± 1.11 *	8.70 ± 0.66
	G_2_/M	40.97 ± 2.73	36.45 ± 0.19 *	32.27 ± 0.38 *
	G_0_/G_1_	41.82 ± 1.63	41.62 ± 2.27	44.05 ± 2.45 *
DP-SC	S	9.91 ± 1.89	12.34 ± 1.26 *	11.63 ± 1.74 *
	G_2_/M	48.25 ±2.47	45.72 ± 3.54 *	43.83± 2.45 *
	G_0_/G_1_	40.36 ± 2.45	42.36± 1.63 *	46.31± 2.27 *
DP-SM	S	11.72 ± 1.74	11.00 ± 1.69	15.84 ± 1.98 *
	G_2_/M	47.91 ± 0.01	46.62 ± 0.53	44.91 ± 0.74 *

Results are expressed as a percentage of total cells. Significant differences between untreated cells (CT) and cells treated with DP-MJ. DP-SC and DP-MA (500–1000 µg/mL) were compared (* *p* < 0.05; ** *p* < 0.01).

**Table 5:** Effect of extracts of dried peel powders (DP) extracts of *M. jaboticaba* (DP-MJ), *S. cumini* (DP-SC), and *S. malaccense* (DP-SM) (500–1000 µg/mL) on programmed cell death in adenocarcinoma cell line after 24 h.

Stages of Cell Death	CT	DP-MJ (µg/mL)		DP-SC (µg/mL)		DP-SM (µg/mL)	
		500	1000	500	1000	500	1000
Viable cells (Annexin V^−^/PI^−^)	95.50 ± 1.25	89.52 ± 0.47 *	87.9 ± 3.00 *	92.8 ± 2.00	91.00 ± 0.80	90.60 ± 0.62	91.95 ± 0.95
Early apoptosis (Annexin V^+^/PI^−^)	2.51 ± 0.78	6.88 ± 0.10 *	7.25 ± 2.72 *	2.27 ± 0.23	2.58 ± 0.22	3.00 ± 0.40	2.80 ± 0.86
Late apoptosis (Annexin V^+^/PI^+^)	1.57 ± 0.85	3.61 ± 0.09 *	4.03 ± 0.13 **	1.08 ± 1.09	0.98 ± 0.40	0.82 ± 0.25	1.27 ± 0.11
Non-apoptotic cells (Annexin V^−^/PI^+^)	4.04 ± 1.08	2.62 ± 0.11 *	1.77 ± 1.14 *	1.84 ± 0.66 *	5.45 ± 1.01	5.60 ± 1.02	3.97 ± 0.97

Results are expressed as a percentage of total cells. Significant differences between untreated cells (CT) and cells treated with DP-MJ, DP-SC, and DP-SM (500–1000 µg/mL) were compared (* *p* < 0.05; ** *p* < 0.01). PI—propidium iodide.

## Data Availability

Not applicable.

## Supplementary Materials

The following are available online, Figure S1: Chromatogram obtained from jabuticaba (Myrciaria jaboticaba, MJ) samples. Peak 1: Delphinidin-3-O-glucoside; peak 2: Cyanidin-3-O-glucoside. Figure S2: Chromatogram obtained from jamun-berry (Syzygium cumini, SC) samples. Peak 1: Delphinidin-3,5-O-diglucoside; peak 2: Cyanidin-3,5-O diglucoside; peak 3: Petunidin-3,5-O-diglucoside; peak 4: Malvidin-3,5-O-diglu-coside. Figure S3: Chromatogram obtained from malay-apple (Syzygium malaccense, SM) sam-ples. Peak 1: Cyanidin-3,5-Odiglucoside; peak 2: Cyanidin-3-O-glucoside.

## Sample Availability

Samples of the compounds are not available from the authors.
