# Supplementary material for: Antiproliferative Activity on Human Colon Adenocarcinoma Cells and In Vitro Antioxidant Effect of Anthocyanin-Rich Extracts from Peels of Species of the Myrtaceae Family
Source: Molecules. 2021 Jan 22;26(3):564. doi: 10.3390/molecules26030564 (PMC7865521; doi:10.3390/molecules26030564)
Supplement: Supplementary file 1 [file molecules-26-00564-s001.pdf]

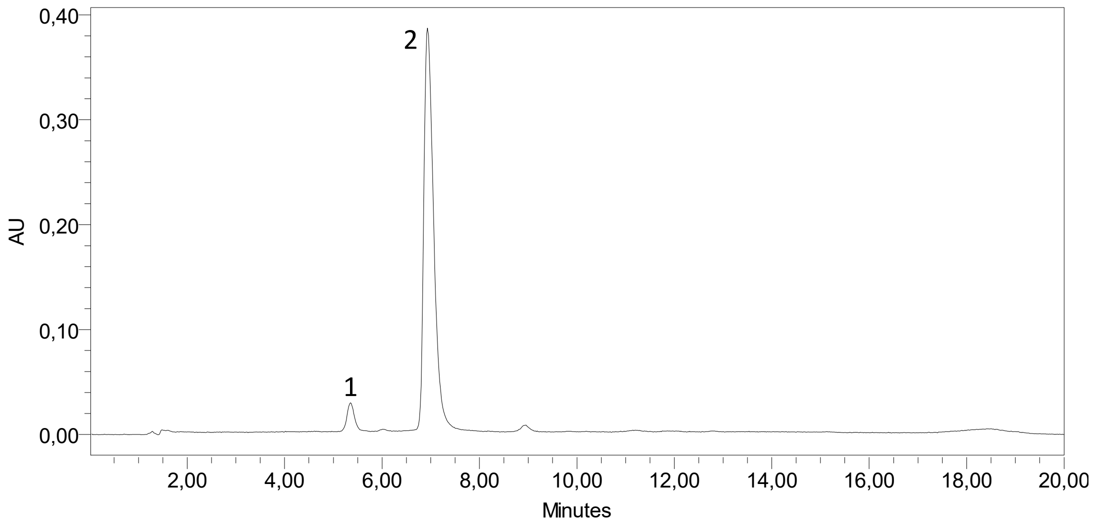

Figure S1. Chromatogram obtained from jabuticaba (*Myrciaria jaboticaba*, MJ) samples. Peak 1: Delphinidin-3-O- glucoside; peak 2: Cyanidin-3-O- glucoside.

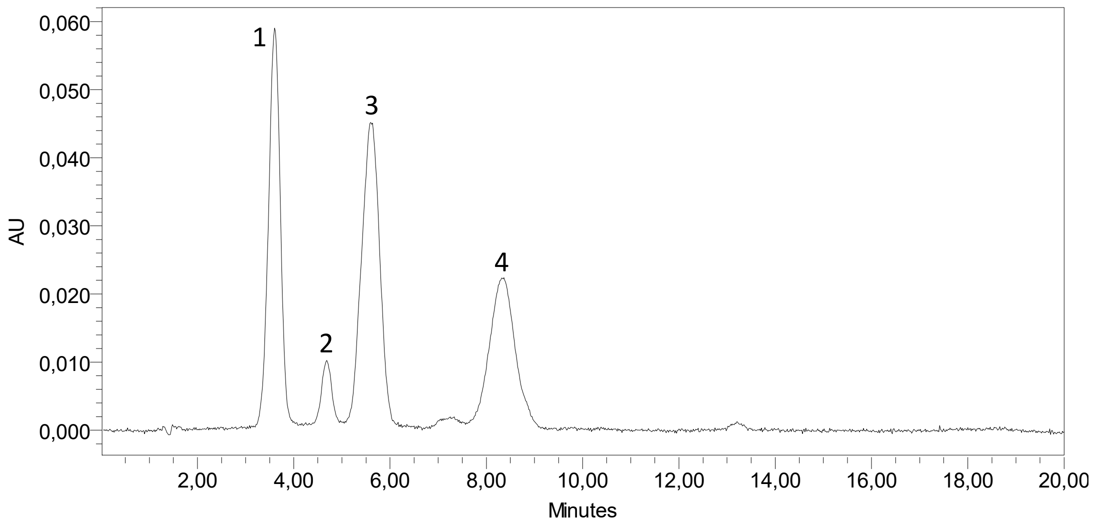

Figure S2. Chromatogram obtained from jamun-berry (*Syzygium cumini*, SC) samples. Peak 1: Delphinidin-3,5-O- diglucoside; peak 2: Cyanidin-3,5-O diglucoside; peak 3: Petunidin-3,5-O-diglucoside; peak 4: Malvidin-3,5-O- diglucoside.

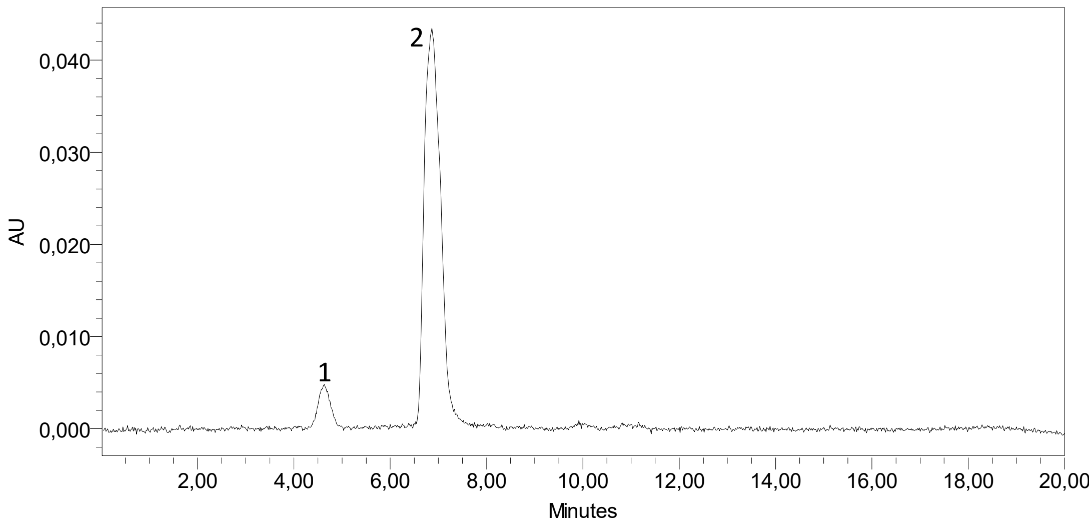

Figure S3. Chromatogram obtained from malay-apple (*Syzygium malaccense*, SM) samples. Peak 1: Cyanidin-3,5-O diglucoside; peak 2: Cyanidin-3-O- glucoside
